# The complete mitochondrial genome of *Scylla paramamosain* (Decapoda: Portunidae)

**DOI:** 10.1080/23802359.2017.1398617

**Published:** 2017-11-07

**Authors:** Shengping Zhong, Yanfei Zhao, Xianfeng Wang, Zhifei Song, Qing Zhang

**Affiliations:** a Key Laboratory of Marine Biotechnology, Guangxi Institute of Oceanology, Beihai, China;; b College of Ocean and Earth Sciences, Xiamen University, Xiamen, China

**Keywords:** Mitochondrial genome, *Scylla paramamosain*, Decapoda

## Abstract

The mud crab *Scylla paramamosain* (Decapoda:Portunidae) is a commercially important crustacean species in China, which is broadly distributed along the southeastern coastal regions of China. In previous studies, the population from Beibu Bay showed distinct genetic lineage. Here, we report the complete mitochondrial genome sequence of *S. paramamosain* from Beibu Bay. The mitogenome has 15,816 base pairs and made up of total of 37 genes (13 protein-coding, 22 transfer RNAs and 2 ribosomal RNAs), and a putative control region. The complete mitogenome in the Beibu Bay population was shorter than that in the Shenzhen population, because the non-coding region between tRNA-Glu and tRNA-His was shorter in the former, and there were 59 mutations sites between these two populations. This study adds a distinct mitogenomes of *S. paramamosain* and will provide useful genetic information for future genetic variation identification and genetic diversity evaluation of this economic valuable crab.

The genus *Scylla* is an economic valuable crustacean known as mud crab, which is an ecologically and commercially important species in the Indo-Pacific region (Imjongjirak et al. 2007). The mud crab, being commercial fisher and increasingly aquafarming in southeast coastal region in China, has become an increasingly important aquaculture species in China (Ma et al. 2013). In previous studies, the cluster analysis of seven wild population of mud crab from southeast coastal region in China showed that Beibu Bay population was an independent cluster, which indicated the population from Beibu Bay was distinct genetic lineage (Shu et al. 2011). Here, we report the complete mitochondrial genome sequence of *S. paramamosain* from Beibu Bay, which will be an important genetic resource to assist in molecular identification of the phylogenetic position between genus *Scylla* and genetic variation within *S. paramamosain*.

The tissue samples of *S. paramamosain* from three individuals was collected from Beibu Bay, China (Beihai, 20°59′21.98″N, 109°2′31.30″E), and the whole body specimens (#GQ0009) were deposited at Marine biological Herbarium, Guangxi Institute of Oceanology, Beihai, China. The total genomic DNA was extracted from the muscle of the specimens using an SQ Tissue DNA Kit (OMEGA, Guangzhou, China) following the manufacturer’s protocol. DNA libraries (350 bp insert) were constructed with the TruSeq NanoTM kit (Illumina, San Diego, CA) and were sequenced (2 × 150 bp paired-end) using HiSeq platform at Novogene Company, China. Mitogenome assembly was performed by MITObim (Hahn et al. 2013). The complete mitogenome of Shenzhen population (GenBank accession no. JX457150.1) was chosen as the initial reference sequence for MITObim assembly. Gene annotation was performed by MITOS (Bernt et al. 2013).

The complete mitogenome from Beibu Bay was found to be 15,816 bp in length (GenBank accession no. MG197997), consisting of the usual set of 13 protein-coding, 22 tRNA and 2 rRNA genes and a putative control region. The overall base composition of the mitogenome was estimated to be A 34.8%, T 38.2%, C 16.9% and G 10.2%, with a high A + T content of 72.9%, which is similar, but slightly different from Shenzhen population (Ma et al. 2013). The complete mitogenome in the Beibu Bay population was shorter than that in the Shenzhen population, because the non-coding region between tRNA-Glu and tRNA-His was shorter in the former, and there were 59 mutations sites between these two populations. This result indicated *S. paramamosain* from Beibu Bay genetically isolated from the other population, which consistent with the similar genetic distinction result of *Apis cerana* (Shinmura et al. 2017). The result of phylogenetic tree of 12 species (including other 12 species from superfamily Portunoidea in NCBI) supported the close relationship between *S. paramamosain* and *S. tranquebarica* (Figure 1), as they shared the same branch node with the highest bootstrap value. COX2 terminated with an incomplete stop codon T, which is thought to be completed with the addition of 3′ adenine residues to the mRNA (Ojala et al. 1981). The complete mitochondrial genome sequence of *S. paramamosain* from Beibu Bay adds a distinct mitogenomes of *S. paramamosain*, which will contribute to further phylogenetic and comparative mitogenome studies of this economic valuable crab.

**Figure 1.**
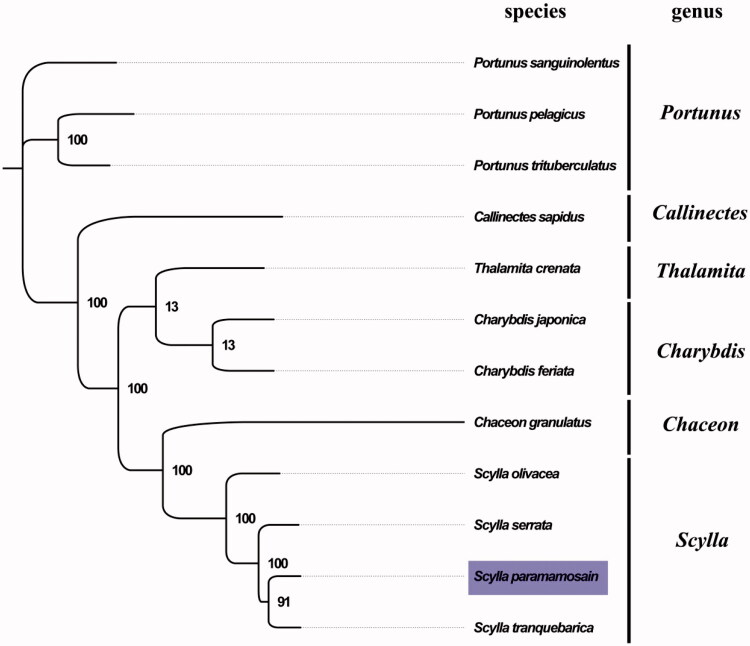
Phylogenetic tree of 12 species in superfamily Portunoidea. The complete mitogenomes is downloaded from GenBank and the phylogenic tree is constructed by maximum-likelihood method with 100 bootstrap replicates. The bootstrap values were labelled at each branch nodes. The gene's accession number for tree construction is listed as follows: *Portunus sanguinolentus* (NC_028225), *P. pelagicus* (NC_026209), *P. trituberculatus* (NC_005037), *Callinectes sapidus* (NC_006281), *Thalamita crenata* (NC_024438), *Charybdis japonica* (NC_013246), *C. feriata* (NC_024632), *Chaceon granulatus* (NC_023476), *S. olivacea* (NC_012569), *S. serrata* (NC_012565), and *S. tranquebarica* (NC_012567).
